# Electrophysiological Properties of Endogenous Single Ca^2+^ Activated Cl^−^ Channels Induced by Local Ca^2+^ Entry in HEK293

**DOI:** 10.3390/ijms22094767

**Published:** 2021-04-30

**Authors:** Dmitrii Kolesnikov, Anastasiia Perevoznikova, Konstantin Gusev, Lyubov Glushankova, Elena Kaznacheyeva, Alexey Shalygin

**Affiliations:** Institute of Cytology, Russian Academy of Sciences, 4 Tikhoretsky Avenue, 194064 St. Petersburg, Russia; koledmi3@mail.ru (D.K.); avp280@gmail.com (A.P.); k.o.gusev@gmail.com (K.G.); glushankova@hotmail.com (L.G.)

**Keywords:** SOCE, CaCC, IP_3_R, TRPC1, anoctamin, TMEM16, single channel, HEK293

## Abstract

Microdomains formed by proteins of endoplasmic reticulum and plasma membrane play a key role in store-operated Ca^2+^ entry (SOCE). Ca^2+^ release through inositol 1,4,5-trisphosphate receptor (IP_3_R) and subsequent Ca^2+^ store depletion activate STIM (stromal interaction molecules) proteins, sensors of intraluminal Ca^2+^, which, in turn, open the Orai channels in plasma membrane. Downstream to this process could be activated TRPC (transient receptor potential-canonical) calcium permeable channels. Using single channel patch-clamp technique we found that a local Ca^2+^ entry through TRPC1 channels activated endogenous Ca^2+^-activated chloride channels (CaCCs) with properties similar to Anoctamin6 (TMEM16F). Our data suggest that their outward rectification is based on the dependence from membrane potential of both the channel conductance and the channel activity: (1) The conductance of active CaCCs highly depends on the transmembrane potential (from 3 pS at negative potentials till 60 pS at positive potentials); (2) their activity (NPo) is enhanced with increasing Ca^2+^ concentration and/or transmembrane potential, conversely lowering of intracellular Ca^2+^ concentration reduced the open state dwell time; (3) CaCC amplitude is only slightly increased by intracellular Ca^2+^ concentration. Experiments with Ca^2+^ buffering by EGTA or BAPTA suggest close local arrangement of functional CaCCs and TRPC1 channels. It is supposed that Ca^2+^-activated chloride channels are involved in Ca^2+^ entry microdomains.

## 1. Introduction

In non-excitable cells, activation of phospholipase C (PLC) mediates the calcium (Ca^2+^) release from the inositol 1,4,5-trisphosphate (IP_3_)-sensitive intracellular Ca^2+^ stores, activation of STIM (stromal interaction molecules) proteins which are sensors of intraluminal Ca^2+^ concentration, and Ca^2+^ influx through the plasma membrane store-operated Orai channels [1,2,3]. Store depletion and Orai channel activation can lead to the activation of TRPC (transient receptor potential-canonical) channels, which amplify and modulate downstream Ca^2+^ signaling [4,5]. There is a dispute whether TRPC1 channels are store-operated per se. On the one hand TRPC1 can be directly regulated by STIM proteins in overexpressed systems [6,7], on the other hand in some cells TRPC1 activation by store depletion requires Ca^2+^ entry through Orai channels [8]. Additionally TRPC1 could be activated in a store-independent manner.

It was shown that in T-lymphocytes store-operated channels have impact on the activity of Ca^2+^-activated chloride channels (CaCCs) and, by this way, cell proliferation [9]. Tissue-specific deletion or loss-of-function mutations in STIM1/2 and/or Orai1 proteins disturbed the functioning of eccrine sweat glands. It was revealed that the mechanism of disturbances in sweat production depends on chloride secretion and activation of CaCCs formed by anoctamin (ANO/TMEM16) [10]. Orai and STIM are also necessary to replenish Ca^2+^ stores in interstitial cells of Cajal and to provide Ca^2+^ release which stimulates CaCCs. Activation of CaCCs, in turn, is involved in slow wave generation and phasic contraction in the gastrointestinal tract [11]. Store depletion activated Ca^2+^ entry through TRPC1 channels is also involved in ANO1 activation in salivary gland cell line [12]. 

Interestingly, proteins involved in calcium-activated chloride entry were shown to influence Ca^2+^ release and Ca^2+^ entry [13,14,15]. Moreover, members of the ANO family can affect Ca^2+^ signaling directly [16]. So, expression of ANO 1, 6, 10 enhances Ca^2+^ signals whereas expression of ANO 4, 8, 9 reduces it [17]. The authors revealed that overexpressed ANO1 colocalizes and interacts with IP_3_Rs, which defines ANO1 activation by Ca^2+^ released from SR, while overexpressed ANO4 interacts with SERCA and Orai1 channels.

Altogether, it is supposed the close localization and interaction between CaCCs and SOCE channels [17,18]. Nevertheless, it remains unclear whether CaCCs are activated by global Ca^2+^ increase in the cytoplasm during SOCE, or if there is a local functional interaction between CaCCs and Orai/TRPC channels.

To investigate this issue, cell-attached and inside-out configurations of patch-clamp recordings of single channels were used. Most of the recent electrophysiological studies used overexpression of ANO, however it is almost impossible to register single channel in overexpression system [19]. Moreover protein overexpression can impact protein arrangement and interaction. Here we studied the endogenous CaCCs of HEK293 (human embryonic kidney) cells in which we previously described and characterized SOCE and TRPC channels [20,21,22]. HEK293 cells are known to expresses endogenous ANO 1, 6, 8, 9 [23,24,25]. Whole-cell experiments showed that relatively high level of intracellular free Ca^2+^ concentration ([Ca^2+^]_i_) in the range of 0.1–1 mM activated Cl-selective endogenous ANO6 current [26]. ANO6 is the dominant CaCC in HEK293 [27,28] and determines their survival [28,29]. However, single-channel properties of endogenous CaCCs are poorly characterized in these cells. 

In this work we characterized the properties of single chloride channels activated by Ca^2+^ entry through TRPC1 in HEK293 cells. Also, we studied the functional interaction between CaCCs and TRPC1 channels by means of “slow” (EGTA) and “fast” (BAPTA) Ca^2+^ chelators.

## 2. Results

### 2.1. IP_3_-Induced Calcium Entry through TRPC1 Activates Outwardly Rectifying CaCCs in HEK293 Cells

**Figure 1:**
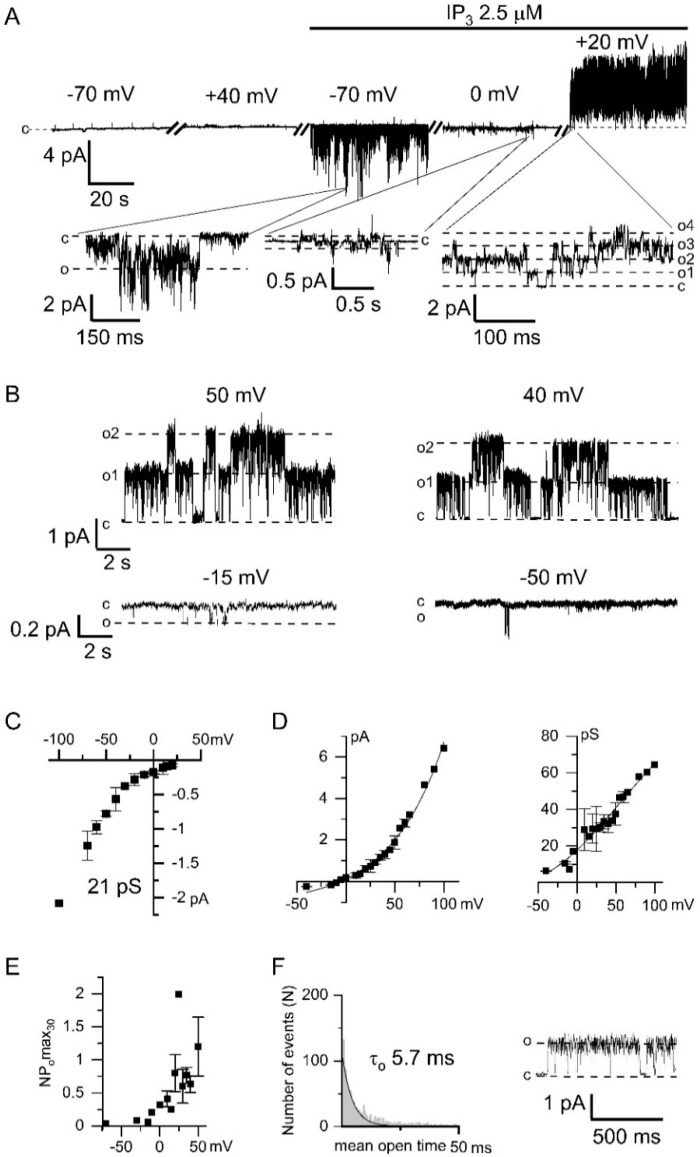
Electrophysiological properties of endogenous Ca^2+^-activated chloride channels (CaCC) in HEK293 cells. (**A**) Representative recording of currents, registered at different membrane potentials, within one experiment before and after bath application of 2.5 μM IP_3_ (inositol 1,4,5-trisphosphate). The expanded panels depict inward IP_3_-induced Ca^2+^ currents followed by Ca^2+^-activated outward currents. The baseline was appropriately adjusted for voltage switches. (**B**) Examples of CaCC current recordings obtained after IP_3_-induced Ca^2+^ entry at different membrane potentials. (**C**) Current–voltage relationship of IP_3_-induced TRPC1 (transient receptor potential-canonical) Ca^2+^ currents (*n* = 3–11). The linear fit to the data points from −100 to −40 mV yielded single channel conductance (γ) of 21 pS. (**D**) *Left*: Current–voltage relationship of Ca^2+^-activated chloride channels recorded in panel B (*n* = 3–4). *Right*: The dependence of the conductance of Ca^2+^-activated chloride channels on the membrane potential. (**E**) The dependence of the activity of Ca^2+^-activated chloride channels, expressed as NPomax30, on the membrane potential (*n* = 4). (**F**) An open-time histogram of CaCC channel was constructed from 2563 single-channel opening events. Single exponential fit (solid line) corresponds to a time constant of 5.7 ± 0.3 ms. The current trace demonstrates a typical fragment of the current recording used for the histogram at +30 mV and filtered at 1 kHz.

The application of 2.5 µM IP_3_ in the inside-out patches of HEK293 cells resulted in the activation of inward calcium channels followed by activation of outward currents pronounced at positive potentials (Figure 1A). The conductance of the calcium channels was approximately 21 pS (Figure 1C). We observed these channels in about 30% of all experiments (*n* = 45). These currents were not observed in experiments without IP_3_ application. In our previous experiments we attributed these currents to TRPC1 channels by means of knockdown and overexpression of TRPC1 protein [20,21]. 

After activation of the Ca^2+^ entry we recorded outgoing currents with large channel amplitude at positive potentials (Figure 1A,B). The current–voltage relationship of these channels had the outward rectification which is characteristic of chloride channels (Figure 1D). There was no CaCC activation at positive potentials before the addition of IP_3_ to the intracellular solution (Figure 1A).

We observed that the single-channel CaCC conductance depends on the membrane potential (Figure 1D). The conductance of the channels at negative potentials was about 7 pS. The CaCC open probability, measured as NPomax30, was increased at positive membrane potentials (Figure 1E). The open lifetime distribution of the chloride channel can be fitted by exponential function with time constant 5.7 ± 0.3 ms (Figure 1F).

**Figure 2:**
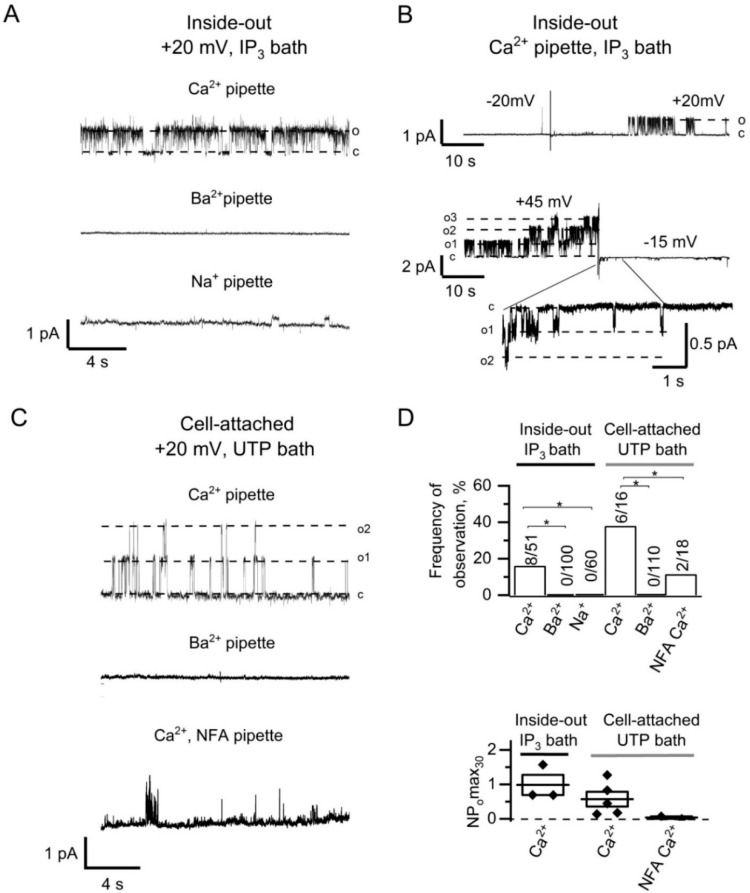
Induction of CaCCs activity by IP_3_-mediated Ca^2+^ entry in HEK293 cells. (**A**) Current recordings after bath application of 2.5 µM IP_3_ to inside-out patches with 105 Ca^2+^ in pipette solution (top), 105 Ba^2+^ in pipette solution (middle), and 140 Na^+^ in pipette solution (bottom). Patches were held at +20 mV membrane potential. (**B**) IP_3_-induced CaCC currents in inside-out patches after switching to positive (top) or to negative (bottom) potential. (**C**) Current recordings after bath application of 100 µM UTP to cell-attached patches with 105 Ca^2+^ in pipette solution (top), 105 Ba^2+^ in pipette solution (middle), and 105 Ca^2+^ in the presence of 100 µM NFA in pipette solution (bottom). (**D**) The summary plot of the frequency of CaCCs observation (top) and the CaCC open channel probability (bottom) for series from the panels A–C. The frequency of CaCCs observation reflects a proportion of positive experiments to the total number of experiments. Differences are considered significant when *p* values are <0.05 (*).

It should be noted that the activity of CaCCs was not observed when the Ca^2+^ was replaced by Ba^2+^ (*n* > 100) or Na^+^ (*n* > 50) in the pipette solution (Figure 2A,D). These data suggest that recorded channels require Ca^2+^ entry and therefore could be attributed to calcium-activated channels.

The channels were activated after switching from negative holding potential to positive potentials, which is consistent with the previously published data for CaCCs [30]. The switch from positive potential to a negative potential resulted in reducing the channel amplitude and in inactivation of the channels (Figure 2B). The initial openings allowed us to distinguish CaCCs at negative potential. 

Subsequent experiments we performed in the cell-attached configuration with pipette and bath Ca^2+^ containing solutions. The application of 100 µM UTP to the bath solution, which leads to the IP_3_ production and induction of Ca^2+^ store depletion activated CaCC currents (Figure 2C). The inclusion of 100 µM niflumic acid (NFA), the chloride channel blocker [24,28], into the Ca^2+^-containing pipette solution reduced both the frequency of observations and the activity of the channels (Figure 2C,D). Also, there was no activation of CaCCs when 105 mM Ba^2+^ was used in the pipette solution in cell-attached recordings (Figure 2C,D). It should be noted that there is 2 mM Ca^2+^ in the bath solution surrounding the cell, which suggest that Ca^2+^ entering through the channels outside the patch area is not sufficient for CaCC activation in the patched area. 

### 2.2. Direct Activation of Endogenous CaCCs in HEK293 by High [Ca^2+^]_i_

**Figure 3:**
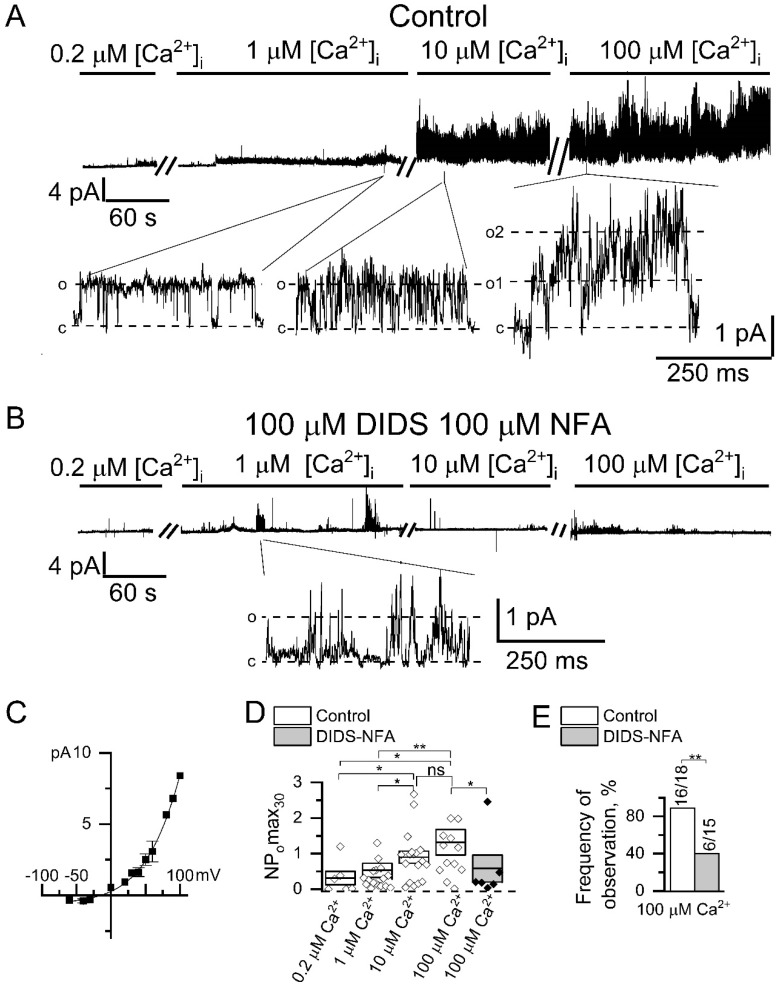
Endogenous CaCCs activated by direct Ca^2+^ application in HEK293 cells. (**A**) Representative recordings of channels activated by the increase in intracellular Ca^2+^ concentration. Shown are traces at compressed and expanded scale recorded in inside-out configuration at holding potential of +40 mV. (**B**) Representative traces of channels activated by the increase in intracellular Ca^2+^ concentration recorded at the same conditions as in panel A but in the presence of 100 µM NFA and 100 µM DIDS in the recording pipette. (**C**) Current–voltage relationship for CaCCs activated by direct 100 µM Ca^2+^ application (*n* = 3). (**D**) A summary plot of the CaCCs open channel probability in inside-out recordings at holding potential of +40 mV. The data are presented for different cytoplasmic Ca^2+^ concentration, and with or without chloride channel blockers NFA (100 µM) and DIDS (100 µM). (**E**) The frequency of CaCC observation in patches is plotted as a proportion of positive experiments to the number of experiments in the series. The data are presented for experiments with bath application of 100 µM Ca^2+^ with or without 100 µM NFA and 100 µM DIDS in the pipette. Data are shown as means ± S.E. Differences are considered significant when *p* values are <0.05 (*), and <0.01 (**).

To ensure that the recorded channels are indeed CaCC, we also performed inside-out experiments with direct activation of the chloride channels by high [Ca^2+^]_i_ (Figure 3). The endogenous CaCCs activation could be induced in most experiments after the application of 1 µM Ca^2+^ in 115 ± 17 s (*n* = 11). Ca^2+^ application activated pronounced channel activity at the concentration above 10 µM (Figure 3A,D).

Channel amplitude was augmented by 28 ± 14% (*n* = 6) after 10 µM Ca^2+^ application and by 15 ± 6% (*n* = 4) after 100 µM Ca^2+^ application. Inclusion of both 100 µM NFA and 100 µM DIDS into the pipette solution significantly suppressed the induction of channel activity activated by high [Ca^2+^]_i_ (Figure 3B,D,E), which confirms that registered channels are chloride channels [31]. Moreover substitution of glutamate with chloride in the intracellular solution shifts reversal potential by 16 ± 2 mV rightward (*n* = 5, Figure S1). The current–voltage relationship of the channels had outward rectification at all used [Ca^2+^]_i_ (Figure 3C). The current–voltage relationship of the activated channels was similar to the current–voltage relationship of the channels, activated through the induction by IP_3_-activated Ca^2+^ channels (Figure 1D). The channel properties described above are similar to previously reported properties of CaCCs ANO6 (TMEM16F) [32,33].

### 2.3. Functional Coupling and Close Arrangement of Endogenous TRPC1 Channels and CaCCs

Based on the data obtained, we can conclude that activation of endogenous CaCCs in HEK293 requires dramatic increase in [Ca^2+^]_i_, which at physiological conditions could be achieved by the Ca^2+^ entry through plasma membrane channels. Moreover, it can be assumed that the activation of CaCCs requires a local Ca^2+^ entry within a close proximity to the channels.

Previously, by means of Ca^2+^ chelator HEDTA, it was shown that in olfactory receptor neurons the distance between cyclic-nucleotide-gated (CNG) channels and CaCCs was estimated within the range of 100–150 nm [34]. In our investigation we applied a similar approach to estimate the functional arrangement of CaCCs and IP_3_-induced calcium channels.

**Figure 4:**
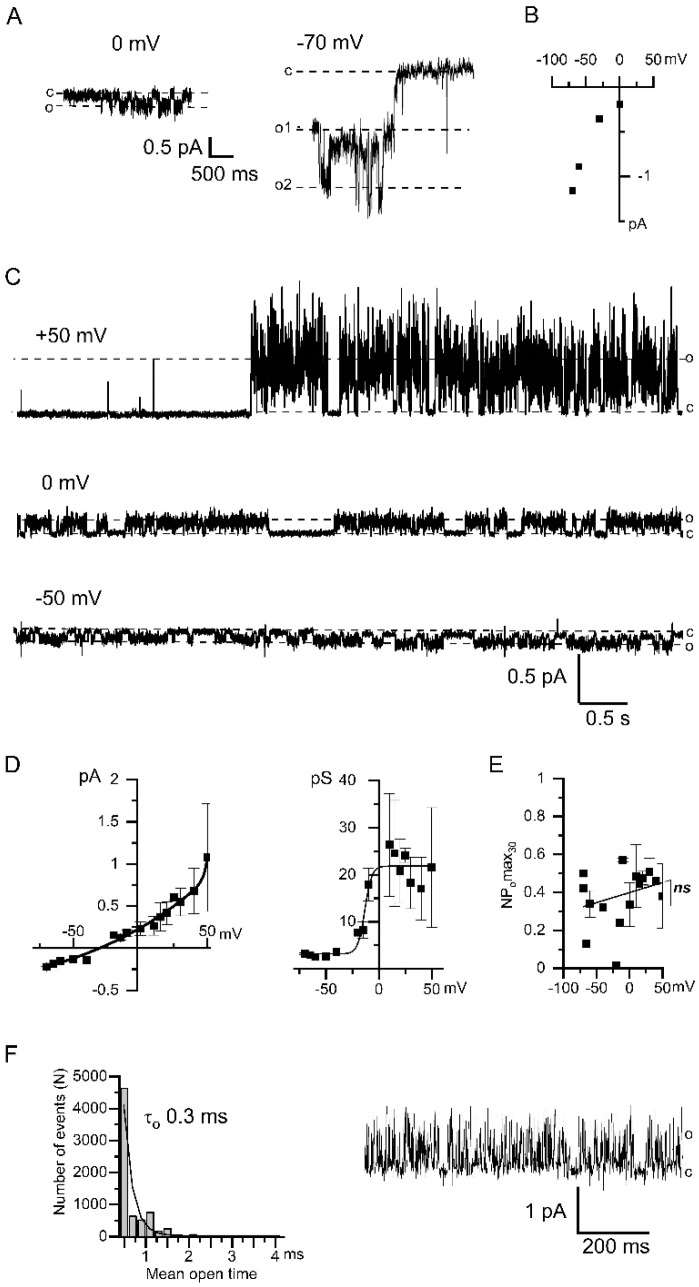
Properties of Ca^2+^-activated chloride channels upon using fast Ca^2+^ chelator 10 mM BAPTA. (**A**) Examples of single channel recordings through the TRPC1 channels induced by the bath application of 2.5 μM IP_3_ in inside-out patches. Membrane potentials were held at 0 mV (left) and −70 mV (right). (**B**) Current–voltage relationship of store-operated TRPC1 channels is induced by 2.5 µM IP_3_ application. Approximation of conductance gives 21 pS (*n* = 3–7). (**C**) Current recordings of CaCCs-activated following IP_3_-induced Ca^2+^ entry registered at different membrane potentials +50 mV, 0 mV, −50 mV (top to bottom). (**D**) *Left*: The current–voltage relationship of CaCCs in the BAPTA containing bath solution (*n* = 4–8). *Right*: The dependence of the CaCC conductance on the membrane potential (*n* = 4–8). (**E**) The dependence of the CaCC activity, expressed as NPomax30, on the membrane potential (*n* = 4). (**F**) Histogram of the open state lifetime distribution of the CaCCs. The membrane potential is +30 mV. An exponential fit gives an average lifetime of 0.3 ± 0.1 ms. The total number of events is *n* = 7946. Typical fragment of the current used for the histogram, monitored at +30 mV and filtered at 1 kHz.

In the inside-out experiments described above (Figure 1 and Figure 2A,B), the [Ca^2+^]_i_ was controlled by 10 mM EGTA, which allows Ca^2+^ ions to diffuse over a distance of about 100 nm [35,36]. In subsequent inside-out experiments, we used an intracellular solution with ~1 nM free Ca^2+^ buffered by 10 mM BAPTA, a faster Ca^2+^ chelator, which limits the effective diffusion of Ca^2+^ ions to 10 nm [35]. In these conditions the addition of IP_3_ led to the activation of the TRPC1 channels (Figure 4A). The current–voltage relationship of TRPC1 channels was not affected by the buffering of Ca^2+^ with BAPTA (Figure 4B). 

In BAPTA-containing intracellular solution Ca^2+^ entry through TRPC1 channels was followed by the activation of CaCCs (Figure 4C). Therefore BAPTA was inefficient to prevent CaCCs activation induced by local Ca^2+^ entry. However, when intracellular Ca^2+^ was buffered at the 1 nM [Ca^2+^]_i_ with BAPTA the CaCCs properties were altered. At potentials negative to the reversal potential, the single-channel conductance of the chloride currents was about 3 pS (Figure 4D). The amplitude of the CaCCs currents registered in the solution with 1 nM [Ca^2+^]_i_ was reduced. The NPomax30 was not dependent on the applied potential (Figure 4E). In BAPTA-containing solutions, the open state of CaCCs appears to be unstable and under-resolved. The estimated lifetime of the channel in the open state was 0.3 ± 0.1 ms (Figure 4F). 

The leftward shift in the reversal potential by −14 mV (Figure 1D and Figure 4D) is apparently associated with the lower concentration of chloride ions used in the intracellular solution with 10 mM BAPTA. Indeed, in accordance with the Goldman-Hodgkin-Katz equation, a decrease in the intracellular chloride concentration from 20 mM to 10 mM shifts the reversal potential by −18 mV. These results provide an additional proof that channels are permeable to chloride ions. 

Since the activity of Ca^2+^-activated chloride channels developed after IP_3_-induced Ca^2+^ entry in the presence of BAPTA in the intracellular solution, it can be concluded that Ca^2+^ was able to diffuse from the pore of the TRPC1 channel to the Ca^2+^-sensitive domain of the CaCCs before interacting with the Ca^2+^ chelator. Based on the results obtained, we assume that the CaCCs are located in close proximity to the TRPC1 plasma membranes channels.

## 3. Discussion

There is no consensus on the characteristics of single CaCC channels [31,37,38]. This is probably related to the very low conductance around 1–3 pS reported in several studies, which makes it difficult to resolve CaCCs at single channel level [38]. Indeed, the registration of single CaCCs with outward rectification at negative potentials is complicated by the low channel amplitudes, which are close to the resolution limit of the device. In order to distinguish the CaCC openings from other activities, we used the amplitude values measured immediately after switching the potential on the membrane from positive to negative values (Figure 2B). In this case, the channel opens with distinct and large amplitude at positive potentials and is clearly resolved at negative potentials, which made it possible to identify it well. 

There are currently no selective CaCCs blockers which would help identify the type of CaCCs. Moreover, the suppression of the gene expression encoding these channels is currently complicated due to the large number of endogenously expressed homologues. Evidence for the identification of single channels is the coincidence of their parameters with the typical properties of the CaCCs described earlier in the whole cell experiments with anoctamin overexpression. Three ANO homologues 1, 2, 6 are forming channels [37]. ANO1 is activated by Ca^2+^ or Ba^2+^ and have single channel conductance up to 8 pS [39]. ANO2 is activated by Ca^2+^, is not activated by Ba^2+^, and its conductance is about 1 pS. ANO1 and ANO2 are activated by less than 1 µM Ca^2+^ [40] and they are inactivated by higher Ca^2+^. ANO1, 2 lose their outward rectification at 3 µM and 13 µM [Ca^2+^]_i_, respectively [41,42].

In this study the registered single channels have quite different properties, which are similar to the typical properties of ANO6, which exhibit outward rectification even at 100 μM Ca^2+^. We observed pronounced activation of CaCCs by [Ca^2+^]_i_ higher than 10 μM, which is also typical for channels formed by ANO6 [43,44,45,46]. Single channel conductance of our registered CaCCs is similar to the previously published conductance of ANO6 about 50 pS [32]. In contrast, another estimation of ANO6 conductance by noise analysis in whole-cell experiments gives 0.45 pS [47]. The reasons for this discrepancy are unknown. Our experiments revealed that the conductance of CaCCs highly depends on transmembrane potential (from 3 pS at negative potentials till 60 pS at positive potentials) and [Ca^2+^]_i_. In our experiments we observed delayed activation of CaCCs at positive potential after increasing of [Ca^2+^]_i_, which is reminiscent to delay in the activation of ANO6 in whole-cell experiments [46]. Previously it was shown that the delay is decreased at higher positive potentials, high [Ca^2+^]_i_, or in the absence of Mg-ATP [44,48]. Based on the channel characteristics we attributed registered CaCCs to ANO6 channels.

Interestingly, some publications demonstrate that ANO6 are anion channels, while other publications describe ANO6 as a non-selective cation channel [24,46,47]. It was shown that ANO6 have a moderate ion selectivity [25], which could be influenced by intracellular Ca^2+^ concentration [33]. In our study the measured reversal potentials of CaCC and the shift in reversal potential by change in intracellular Cl^−^ concentration (Figure 1, Figure 3, Figure 4 and Figure S1) assume that observed channels are predominantly permeable for Cl^−^ ions with slight permeability for other ions used in our solutions.

[Ca^2+^]_i_ mostly modified CaCC activity and only slightly changed single channel amplitude (Figure 3). Decrease of CaCCs whole-cell currents at low [Ca^2+^]_i_ [49] could be explained by our results with Ca^2+^ buffering by BAPTA, which primarily reduces dwell time of open state CaCCs and therefore open probability of channels (Figure 4G,H). 

Our data provide explanation of outward rectification previously observed in whole-cell recordings of CaCCs [50]. Both the conductance and the open probability of CaCCs increased at positive potentials (Figure 1). With lowering Ca^2+^ concentration this dependence disappeared, while NPo and conductance properties became constant. However, channel keeps asymmetric behavior with two different conductances for inward and outward chloride current (Figure 4).

The voltage modulation of the CaCC activity is determined by the voltage-dependent occupancy of Ca^2+^-binding sites located on the sixth transmembrane domain of ANO [33,51]. Most of ion channels have relatively constant single channel conductance at different membrane potentials. The dependence of channel conductance from voltage was described for OmpF porins [52]. Pore electrostatic field affects the channel’s permeability in OmpF porins and ANO6 [52,53]. 

Our data with single channel activity recordings are in agreement with the hypothesis of wide vestibule for Ca^2+^ in ANO6, where Ca^2+^ could dwell for a prolonged time [33]. We assume that after initial rise in intracellular [Ca^2+^] and membrane depolarization, Ca^2+^ ions could reside for prolonged time (minutes) inside the channel, promoting its activity. It is likely that second Ca^2+^-binding site is quickly modulated by cytoplasmic Ca^2+^ or transmembrane potential. 

CaCC properties unequally depend on membrane potential and [Ca^2+^]_i_. The conductance of CaCCs rises by an order of magnitude in the range from −50 to +100 mV (Figure 1D). The CaCCs activity is enhanced by 4–6 times with increasing [Ca^2+^]_i_ and/or transmembrane potential (Figure 1E and Figure 3D). The CaCC amplitude is increased by ~1.5 times with augmentation of [Ca^2+^]_i_ from 1 to 100 μM (Figure 3D).

There are various mechanisms that increase [Ca^2+^]_i_ to the level required for the CaCC activation [31]. Particularly, CaCCs can be activated by the Ca^2+^ entry through voltage-gated Ca^2+^ channels. However, we used non-excitable HEK293 cells and, moreover, in our experiments changing the potential by itself, without adding IP_3_, did not activate the channels. CaCCs formed by ANO2 proteins, can be activated by the Ca^2+^ released through IP_3_R [15]. Our experiments with replacing the Ca^2+^ to Ba^2+^ or Na^+^ in pipette solution showed necessity of Ca^2+^ entry for activation of endogenous CaCCs in HEK293 cells (Figure 2A,C,D). 

It is known that after the Ca^2+^ channel opening, a steady-state [Ca^2+^]_i_ gradient is rapidly established. This gradient is decreased with distance from the channel pore and determined by the buffer properties of the medium [54]. This fact allows to estimate the distance between the Ca^2+^ entry site and Ca^2+^-dependent channels. So, using the Ca^2+^ buffer HEDTA, the distance between the CNG and CaCCs gives a value of about 120 nm [34]. Most of the previous studies investigated the relationship between SOCE channels and CaCCs using macro methods reflecting an increase in [Ca^2+^]_i_ in the entire cytoplasm [10,17]. Colocalization and co-immunoprecipitation of SOCE channels and CaCC components have been previously shown [17], but these data also do not directly prove the functional coupling of the colocalized channels. In contrast, an elegant study demonstrated that SOCE- and IP_3_-induced CaCC activity is spatially separated but functionally coupled by Ca^2+^ tunneling in oocytes [55]. In our experiments, even the fast and strong Ca^2+^ chelator BAPTA could not prevent the activation of CaCC channels, which suggests a very close distance, on the order of 10 nm [35], between the Ca^2+^ influx channel and the chloride channel. In oocytes BAPTA precluded CaCC activity induced by calcium entry evoked by store depletion with IP_3_ [55]. Moreover, in our cell-attached experiments, distant calcium entry, outside the pipette, was not able to activate CaCC in the area under the pipette (more than 100 nm) in contrast to oocytes where Ca^2+^ travels at least to 1 μm. Therefore localization of CaCCs and SOCE in HEK293 cells differs from oocytes. SOCE could increase calcium concentration to more than >20 μM in the vicinity of the channel because chelators are not fast enough to bind it [56]. It is known that ANO6 is activated by [Ca^2+^]_i_ above 10 μM [24]. It can be assumed that in physiological conditions, this high [Ca^2+^]_i_ can be achieved by close proximity of ANO6 to TRPC1 channels. Previously, we demonstrated that TRPC1 channels are involved in SOCE in HEK 293 cells [20,21].

ANO6 is a phospholipid scramblase and ion channel [45]. Disturbances in its activity can result in pathologies of blood coagulation [47], bone formation [57,58], immune system [59]. On the other hand, the same pathologies are caused by disturbances in the activity of store-operated Ca^2+^ channels [60]. That is additional evidence of functional coupling between ANO6 and SOCE. 

In this study we have investigated single Ca^2+^-dependent chloride channels in HEK293 cells. We demonstrated that their amplitude and open probability are increased at positive potentials on the plasma membrane, while a decrease in [Ca^2+^]_i_ leads to the reduction of the channel dwell time in the open state and open probability. We showed the close proximity and functional coupling of TRPC1 channels and Ca^2+^-activated chloride channels, which apparently determine their mutual influence on each other.

## 4. Materials and Methods

### 4.1. Cells

Human embryonic kidney cells (HEK293, Cell Culture Collection, Institute of Cytology, St. Petersburg, Russia) were cultured in DMEM with 10% FBS, 100 µg/mL penicillin, 100 U/mL streptomycin in CO_2_-incubator at 37 °C. Cells were seeded on the glass coverslips covered by polylysine and maintained in culture for 3–4 days before experiments.

### 4.2. Electrophysiological Analysis

Registration of single channels was performed by patch-clamp technique in inside-out and cell-attached modes.

Micropipettes were done from glass filaments Sutter BF150-86-10 and coated by Sylgard for low-noise recordings. The pipettes had resistance 7–12 MOhm, and the gigaseal between cell and micropipette with resistance more than 20 GOhm were used for recordings. The liquid junction potential was not corrected.

Several pipette solutions were used and contained (in mM): (1) 105 CaCl_2_ (in some experiments 105 BaCl_2_/140NaCl was used) 10 Tris-HCl, pH 7.2; (2) 2 CaCl_2_, 145 Tris-HCl, pH 7.2.

The intracellular solution for inside-out recordings contained (in mM): 133 CsGlutamate, 5 MgCl_2_, 1 MgATP, 10 BAPTA, 10 HEPES pH 7.3 (free Ca^2+^ <1 nM, or pCa9, according to MaxChelator; Figure 4) or 130 CsGlutamate, 5 CaCl_2_, 5 MgCl_2_, 1 MgATP, 10 EGTA, 10 HEPES pH 7.2 (free Ca^2+^ ~200 nM, or pCa 6.7, according to MaxChelator; Figure 1 and Figure 2) for 1 µM Ca^2+^ 8.5 mM CaCl_2_ was added, for 10 µM Ca^2+^ 9.82 mM CaCl_2_ was added, in solution with 100 µM Ca^2+^ was added 0.1 mM CaCl_2_ and EGTA was omitted (Figure 3 and Figure S1).

The extracellular solution for cell-attached recordings contained (in mM): 140 KCl, 5 NaCl, 10 K-HEPES, 1 MgCl_2_, 2 CaCl_2_, pH 7.4.

Ca^2+^ entry required for the activation of Ca^2+^-activated chloride channels was induced by adding 2.5 µM IP_3_ to the intracellular solution (unless otherwise indicated) at a maintained potential on the membrane of −70 mV.

All chemicals were from Sigma-Aldrich.

An Axopatch 200B amplifier with a built-in 1 kHz filter was used to register the currents; the data were digitized at a frequency of 5 kHz using a Digidata 1322A. Additional filtering of 100 Hz was used to represent the data (except for cases where the filter frequency is indicated separately).

To assess the activity of the channels, the value NPomax30 = I/i was used, where “I” is the average current measured within a 30-s interval, when the activity of the channels was maximum, and “i” is the current amplitude of a single channel at a given potential. 

The data were processed using the pClamp 10.4 (Molecular Devices) and OriginPro 2018 (OriginLab) software. 

### 4.3. Statistical Analysis

Statistical comparisons were performed either with pairwise Mann–Whitney analysis or with Boschloo’s test for discrete values. Data are shown as means ± S.E. Differences are considered significant when *p* values are <0.05 (*), and <0.01(**).

## Data Availability

The data presented in this study are available on reasonable request from the corresponding author.

## Supplementary Materials

The following are available online at https://www.mdpi.com/article/10.3390/ijms22094767/s1.
