# Supplementary material for: Electrophysiological Properties of Endogenous Single Ca2+ Activated Cl− Channels Induced by Local Ca2+ Entry in HEK293
Source: Int J Mol Sci. 2021 Apr 30;22(9):4767. doi: 10.3390/ijms22094767 (PMC8124839; doi:10.3390/ijms22094767)
Supplement: Supplementary file 1 [file ijms-22-04767-s001.zip › ijms-1190383-supplementary.pdf]

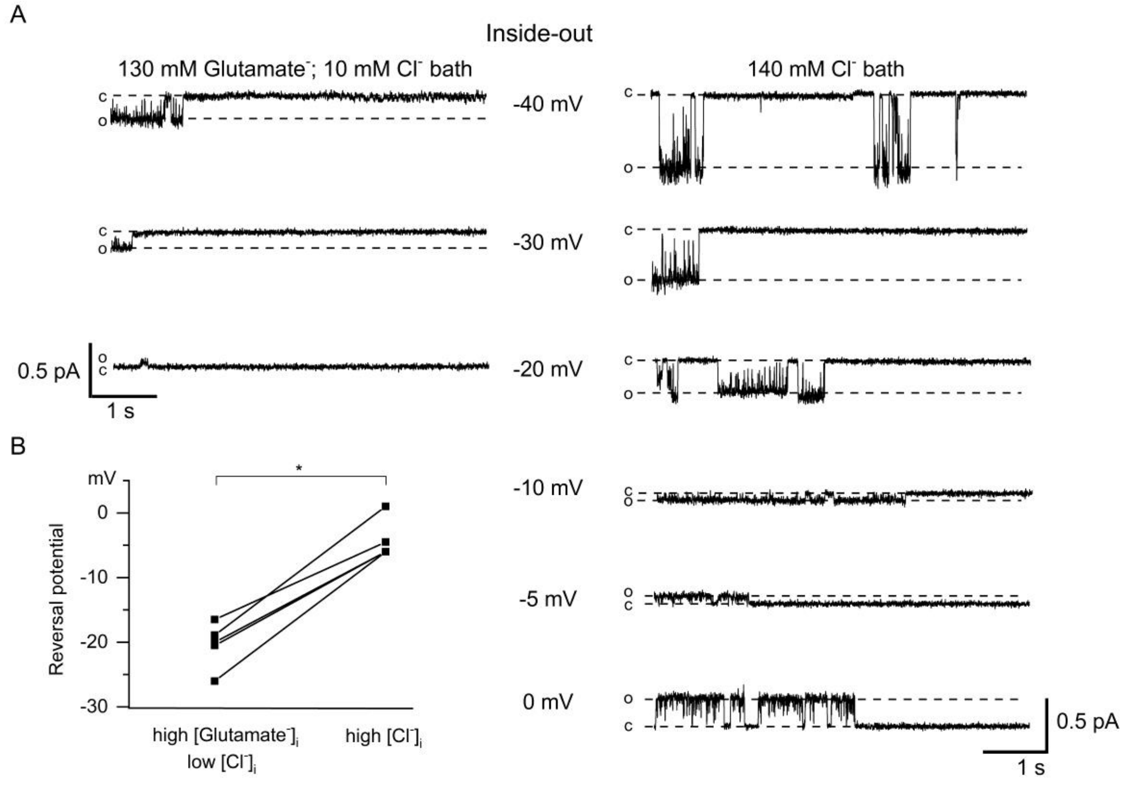

Supplementary Figure S1. Alteration in intracellular chloride concentration leads to shift in CaCC's reversal potential. Current recordings were performed in inside-out patches with 105 mM $CaCl_2$ in the pipette. The channel activity was induced by addition of 100 μM Ca^{2+} to the bath. (A) Anion currents via CaCC channel before and after application of bath solution with higher $[Cl^-]_i$ at membrane potentials as indicated. *Left* – current recordings in 130 mM Glutamate⁻, 10 mM Cl^- in bath solution. *Right* – current recordings in 140 mM Cl^- in bath solution. (B) A summary graph demonstrating reversal potential shift of CaCCs before and after application of bath solution with higher $[Cl^-]_i$ (n = 5); *$p < 0.05$ by the Paired-Sample Wilcoxon Signed Rank Test.
